# Gardens about Hospitals

**Published:** 1918-09

**Authors:** 


### Gardens About Hospitals.

The Government is finding that much advantage comes from keeping the convalescent busy, that restoration to health comes much quicker where the patient's mind and body are kept as active as physical condition will permit. This same thought has occurred to those in charge of hospitals in this country, but they have contented themselves with allowing the convalescents merely to admire the beautiful grounds without busying themselves with their keeping. Acres and acres of gardens located near the American base hospitals are now beginning to bring forth their harvests of vegetables. All were cultivated by American wounded, convalescents, who, during their period of repose, were able to attend to the growing of the produce.

Not alone has the venture been one of great economy for the hospitals. The work of cultivation has been placed upon the prescriptions of the medical surgeons so that a double purpose is accomplished in the establishment of the gardens. The garden work provides relaxation for the men after their strenuous times on the battlefront and their painful anxiety in the hospital wards. The diversion acts as a recreative agency in bringing back the men to normal mental and physical state.

The gardens are worked entirely by convalescents. Not a single dollar has been spent for hired labor. The commanding officers of the varied base hospital units have spoken in lavish terms of the physical good to the men and of the economy and efficacy of the plan, which insures fresh luscious vegetables to the patients at a minium cost to the government.

The army has undertaken a comprehensive plan of army provisioning along these lines. The American Red Cross also is cooperating by the establishment of smaller plots in the centers where convalescents are sent.

